# Startle myoclonus induced by Lyme neuroborreliosis: a case report

**DOI:** 10.1186/1752-1947-7-124

**Published:** 2013-05-13

**Authors:** Julia Schoof, Christian Kluge, Hans-Jochen Heinze, Imke Galazky

**Affiliations:** 1 Department of Neurology, Otto-von-Guericke-University, Leipziger Strasse 44, Magdeburg, 39120, Germany

## Abstract

**Introduction:**

The normal startle response is a form of physiological myoclonus. Its anatomic origin is probably the brain stem. Pathologic startles are defined as reproducible exaggerated startle responses to trivial and not surprising stimuli. Symptomatic forms of an exaggerated startle response can be due to a variety of brain stem disorders. We have, however, found scant data about an exaggerated startle reflex induced by Lyme neuroborreliosis. We therefore report the case of a patient with this unusual presentation.

**Case presentation:**

A 69-year old Caucasian man presented with a two-week history of a pronounced startle myoclonus, as well as a four-week history of double vision, gait disturbance and severe lancinating pain in his upper thoracic region. Neurological examination showed an excessive startle reaction of his upper trunk evoked by visual and tactile stimulation, a positive sign of Lhermitte, mild right-sided palsy of his sixth and seventh cranial nerve, moderate dysarthria, very brisk deep tendon reflexes, pallhypesthesia of his legs, and an atactic gait disturbance. A diagnosis of a Lyme neuroborreliosis was confirmed by cerebrospinal fluid examination. Under intravenous treatment with ceftriaxone, our patient improved considerably with complete remission in a follow-up at two months.

**Conclusions:**

This case illustrates the chameleon role that neuroborreliosis likes to play: although the wide spectrum of different symptoms that neuroborreliosis can present with has been described, to the best of our knowledge this is the first case report about a symptomatic form of a pathologic startle response as the predominating sign of Lyme neuroborreliosis.

## Introduction

The normal startle response is a form of physiological myoclonus. Its clinical presentation usually consists of an involuntary jerky movement (with blink, contortion of the face, flexion of neck and trunk, and abduction and flexion of the arms) evoked by a sudden and unexpected acoustic stimulus. Its anatomic origin is probably the brain stem [1]. Pathologic startles are defined as reproducible exaggerated startle responses to trivial and not surprising stimuli [2]. The most common form of an exaggerated startle response is hereditary hyperekplexia, which is genetically characterized by defects in different gene families involved in glycine neurotransmission [3]. Symptomatic forms of an exaggerated startle response are less frequent and can be due to a variety of brain stem disorders, tetanus, strychnine intoxication or stiff-man syndrome [1,2,4]. We have, however, found scant data about an exaggerated startle reflex induced by Lyme neuroborreliosis [5]. We therefore report the case of a patient with this unusual presentation.

## Case presentation

A 69-year old Caucasian man presented with a two-week history of pronounced startle myoclonus elicited by unexpected visual or tactile stimuli to his upper body, which had led to a fall shortly before admission. In addition, he had a four-week history of double vision, gait disturbance and severe lancinating pain in his upper thoracic region. His medical history included arterial hypertension, a mild left-sided stroke due to a lacunar infarction of the basal ganglia six years ago and severe bilateral hypacusis requiring hearing aids. On admission, a neurological examination showed an excessive startle reaction of his upper trunk evoked by visual and tactile stimulation of the mantle region, positive sign of Lhermitte, mild right-sided palsy of his sixth and seventh cranial nerves, moderate dysarthria, pre-existing latent hemiparesis of his left side, irregular moderate postural tremor of both hands, mild dysmetria of both legs, very brisk deep tendon reflexes, pallhypesthesia of his legs, and an atactic gait disturbance.

A magnetic resonance imaging (MRI) scan of his head showed diffuse unspecific signal alterations compatible with pre-existing vascular lesions. An MRI scan of his cervical spine demonstrated no pathology except for degenerative alterations of his cervical column. Results of an electroencephalogram were normal. His cerebrospinal fluid (CSF) showed a predominantly lymphocytic pleocytosis (500 cells/μL), a raised protein level (2370mg/L) and a positive immunoglobulin titer for *Borrelia burgdorferi* with a specific antibody index (CSF/serum) of 8.2 for immunoglobulin G and 4.0 for immunoglobulin M (normal value <0.300) [6]. All the other neurotropic virologic and luetic antibody titers were negative. Thus, a diagnosis of a Lyme neuroborreliosis was confirmed.

Our patient improved considerably after a few days of an intravenous treatment with ceftriaxone of 2g per day, which was continued for two weeks. His lancinating pain syndrome, cranial nerve palsies and atactic gait disappeared. His exaggerated startle reaction to visual and tactile stimuli rapidly diminished. On discharge, the only remaining symptom was a clinically irrelevant myoclonus of his right sternocleidomastoid muscle after a tactile stimulus of the vertex. Two months later, our patient had completely recovered. The lymphocytic pleocytosis of his CSF had dropped to 11 cells/μL (Additional file 1).

## Discussion

Although there are many reports about symptomatic pathologic startle reactions, we have found scant data about an exaggerated startle reflex induced by Lyme neuroborreliosis [5]. In the case we describe here, our patient presented with some typical signs of Lyme neuroborreliosis, including Bannwarth’s syndrome, a meningoradiculitis that presented with lymphocytic pleocytosis in his CSF; cranial nerve palsies (VI and VII on his right side); and radicular thoracic pain. Nevertheless, such a considerable startle response to tactile and visual stimuli is uncommon in this disease. The lack of startle reaction to acoustic stimuli could be explained by the severe bilateral hypacusis. The brain stem is thought to generate the startle reflex [1], thus brain stem involvement can be assumed in our patient’s case of neuroborreliosis, even though MRI showed no relevant pathology in that area.

## Conclusions

This case further illustrates the chameleon role that neuroborreliosis likes to play: although the wide spectrum of different symptoms that neuroborreliosis can present with has been described, to the best of our knowledge this is the first case report about a symptomatic form of a pathologic startle response as the predominating sign of Lyme neuroborreliosis.

## Consent

Written informed consent was obtained from the patient for publication of this case report and accompanying images. A copy of the written consent is available for review by the Editor-in-Chief of this journal.

## Abbreviations

CSF: Cerebrospinal fluid; MRI: Magnetic resonance imaging.

## Competing interests

The authors declare that they have no competing interests.

## Authors’ contributions

JS and IG were responsible for the stratification and treatment of the patient and the interpretation of the clinical data. JS and IG drafted the manuscript. HJ-H and CK critically revised the manuscript. All authors have read and approved the final version of this manuscript.

## Supplementary Material

Additional file 1 **Startle response.** Segment 1. In the first diagnostic test, the intention to tap the left arm (the visual stimulus) elicits a startle response that mainly consists of bilateral flexion of his arms. His legs are not involved. In the subsequent diagnostic tests, there is no reaction after a visual stimulus but a non-habituating startle reaction after tactile stimulation on his left arm. Segment 2. Seven days after antibiotic treatment, there is no startle response after tactile stimulation on his chest or arms. However, a myoclonic response of the right sternocleidoid muscle after a gentle tap on the vertex can still be observed, which was no longer seen in the follow-up at two months. Click here for file
